# Supplementary material for: The Systems Biology Research Tool: evolvable open-source software
Source: BMC Syst Biol. 2008 Jun 29;2:55. doi: 10.1186/1752-0509-2-55 (PMC2446383; doi:10.1186/1752-0509-2-55)
Supplement: Additional file 1 — SBRT Archive. An archive of the current version of the Systems Biology Research Tool. [file 1752-0509-2-55-S1.zip › sbrt-1.4.0/doc/developers_guide/api/sbrt/kernel/math/geom/comp/CdHarComputer.Results.html]

CdHarComputer.Results


|  |  |  |
| --- | --- | --- |
| **Overview** \| **Package** \| **Class** \| **Use** \| **Tree** \| **Deprecated** \| **Index** \| **Help** | |  |
| **PREV CLASS**   **NEXT CLASS** | **FRAMES**    **NO FRAMES**     **All Classes** |  |
| SUMMARY: NESTED \| FIELD \| CONSTR \| METHOD | DETAIL: FIELD \| CONSTR \| METHOD |  |


---


## sbrt.kernel.math.geom.comp Class CdHarComputer.Results

```
java.lang.Object
  sbrt.kernel.math.geom.comp.CdHarComputer.Results
```

**Enclosing class:**: CdHarComputer<V>

---

``` public class CdHarComputer.Results extends java.lang.Object ```

This class is used to represent the results of a random point
computation.

**Author:**
:   This class was written and documented by Jeremiah Wright while in
    the Wagner lab.

---

| **Method Summary** | |
| --- | --- |
| `int` | `changedVariables()`             Returns the number of variables whose values are different than they were in the previous point. |
| `java.util.Set<V>` | `getChangedVariables()`             Returns the variables whose values are different than they were in the previous point. |
| `UnmodifiableMap<V,java.lang.Double>` | `getPoint()`             Returns the random interior point that was computed. |
| `UnmodifiableSet<V>` | `getUnchangedVariables()`             Returns the variables whose values are the same as they were in the previous point. |
| `int` | `unchangedVariables()`             Returns the number of variables whose values are the same as they were in the previous point. |

**Methods inherited from class java.lang.Object**

`clone, equals, finalize, getClass, hashCode, notify, notifyAll, toString, wait, wait, wait`

**Method Detail**

### getPoint

```
public UnmodifiableMap<V,java.lang.Double> getPoint()
```

:   Returns the random interior point that was computed.

    :   **Returns:**: the random interior point that was computed.

---


### getChangedVariables

```
public java.util.Set<V> getChangedVariables()
```

:   Returns the variables whose values are different than they were in
    the previous point. Ideally, all of the variables of the polytope are
    present in this set.

    :   **Returns:**: the variables whose values are different than they were in the previous point.

---


### getUnchangedVariables

```
public UnmodifiableSet<V> getUnchangedVariables()
```

:   Returns the variables whose values are the same as they were in the
    previous point. Ideally, this set should be empty. If it isn't, it
    could mean the random walker is stuck in a corner.

    :   **Returns:**: the variables whose values are the same as they were in the previous point.

---


### changedVariables

```
public int changedVariables()
```

:   Returns the number of variables whose values are different than they
    were in the previous point. Ideally, this number should equal the
    total number of variables in the convex polytope.

    :   **Returns:**: the number of variables whose values are different than they were in the previous point.

---


### unchangedVariables

```
public int unchangedVariables()
```

:   Returns the number of variables whose values are the same as they
    were in the previous point. Ideally, this number should equal 0.

    :   **Returns:**: the number of variables whose values are the same as they were in the previous point.


---


|  |  |  |
| --- | --- | --- |
| **Overview** \| **Package** \| **Class** \| **Use** \| **Tree** \| **Deprecated** \| **Index** \| **Help** | |  |
| **PREV CLASS**   **NEXT CLASS** | **FRAMES**    **NO FRAMES**     **All Classes** |  |
| SUMMARY: NESTED \| FIELD \| CONSTR \| METHOD | DETAIL: FIELD \| CONSTR \| METHOD |  |


---
